# Comparison between MICRO–CARD–FISH and 16S rRNA gene clone libraries to assess the active versus total bacterial community in the coastal Arctic

**DOI:** 10.1111/1758-2229.12013

**Published:** 2012-12-20

**Authors:** Daniele De Corte, Eva Sintes, Taichi Yokokawa, Gerhard J Herndl

**Affiliations:** 1 Department of Biological Oceanography, Royal Netherlands Institute for Sea Research (NIOZ)PO Box 59, 1790 AB, Den Burg, The Netherlands; 2 Center for Ecological and Evolutionary Studies, University of GroningenPO Box 11103, NL-9700 CC, Groningen, The Netherlands; 3 Department Marine Biology, Faculty Center of Ecology, University of ViennaAlthanstrasse 14, A-1090, Vienna, Austria

## Abstract

We collected surface- and deep-water samples (maximum depth 300 m) during the spring–summer transition in the coastal Arctic along a transect in the Kongsfjorden (Ny-Ålesund, Spitsbergen, Norway) to determine the structure of the active versus total marine bacterioplankton community using different approaches. Catalysed reporter deposition–fluorescence *in situ* hybridization combined with microautoradiography (MICRO–CARD–FISH) was used to determine the abundance and activity of different bacterial groups. The bacterial communities were dominated by members of Alphaproteobacteria followed by Bacteroidetes, whereas Gammaproteobacteria were present at low abundance but exhibited a high percentage of active cells taking up leucine. The clone libraries of 16S rRNA genes (16S rDNA) and 16S rRNA from two different depths were used to decipher the bacterial community structure. Independently of the type of clone libraries analysed (16S rDNA- or 16S rRNA-based), four major and four minor taxonomic groups were detected. The bacterioplankton community was mainly dominated at both the DNA and the RNA levels by Alphaproteobacteria followed by Gammaproteobacteria. The Rhodobacteriaceae were the most abundant members of the Alphaproteobacteria in both DNA and RNA clone libraries, followed by the SAR11 clade, which was only detectable at the 16S rDNA level. Moreover, there was a general agreement between the results obtained with both techniques, although some specific phylogenetic groups, such as SAR11 and Roseobacter, deviated substantially from this relation. These discrepancies are most likely linked to different physiological states among members of the bacterioplankton community. Combined, MICRO–CARD–FISH and DNA and RNA clone libraries, however, allowed for accurately quantifying different bacterial groups and their activity as well as a detailed phylogenetic insight into the fractions of present versus metabolically active bacterial groups.

## Introduction

During the spring to summer transition period, the coastal Arctic is characterized by increasing temperatures, large input of freshwater originating from the adjacent glaciers and melting snow, and a considerable load of terrigenous particles transported via creeks into the coastal regions. Under these conditions, phytoplankton blooms and subsequently bacterial abundance and production are stimulated (Hasle and Heimdal, 1998; Owrid *et al*., 2000; Schoemann *et al*., 2005; Piwosz *et al*., 2009). The bacterial community in the Arctic Ocean consists, like in other marine waters, of few abundant and a large number of rare phylotypes, most of them with unknown ecological functions in the biogeochemical cycling (Pedros-Alio, 2006; Galand *et al*., 2009; Kirchman *et al*., 2010).

Over the last two decades, several molecular techniques based on 16S rDNA such as ARISA (automated ribosomal intergenic spacer analysis), T-RFLP (terminal restriction fragment length polymorphism), DGGE (denaturing gradient gel electrophoresis), cloning and (pyro)sequencing have been used to characterize the complexity of bacterioplankton communities (Muyzer and Smalla, 1998; Fisher and Triplett, 1999; Moeseneder *et al*., 1999; Sogin *et al*., 2006; Pommier *et al*., 2007). However, these techniques do not necessarily reflect the structure of the active microbial community (Moeseneder *et al*., 2005). The total DNA pool of a bacterial community might consist of DNA derived from living, dormant or even dead cells and extracellular DNA (Josephson *et al*., 1993). In contrast to DNA, RNA has a much shorter life span and can serve as an indicator of the metabolically active fraction of the community (Mills *et al*., 2005; Moeseneder *et al*., 2005; Gentile *et al*., 2006). Therefore, there is a linear relation between the rRNA content and the growth rate in bacteria (Delong *et al*., 1989; Kemp *et al*., 1993; Kerkhof and Ward, 1993). Hence, during starvation, the rRNA content decreases to minimum levels in the cell (Fegatella *et al*., 1998). The higher amount of rRNA in active than in dormant cells associated with the higher number of ribosome in active cells provides a tool to determine the metabolically active members of the bacterial community (Poulsen *et al*., 1993). The detection of bacteria on the 16S rDNA level is mainly depending on the abundance of the specific bacterial organism or group in the environment. Bacterial organisms present at low abundance and therefore not detectable at the DNA level might be still detectable at the RNA level if they are metabolically active and thus have a higher ribosome content (Moeseneder *et al*., 2005). Consequently, the comparison between DNA and RNA clone libraries can contribute to understanding the ecological role of the low-abundance phylotypes and might provide insights into community changes related to changing environmental conditions.

One widely used approach to enumerate the active bacterial community is the combination of microautoradiography and fluorescence *in situ* hybridization (Lee *et al*., 1999). This method allows the identification and quantification of specific target prokaryotic groups as well as the uptake of specific radiolabelled substrates. One limitation of this method is that it requires a minimum abundance and uptake rates of the target group in the sample (Teira *et al*., 2004).

In this study, a comparative approach was taken using MICRO–CARD–FISH and 16S rDNA and 16S rRNA clone libraries to resolve the dynamics in the active versus total bacterial community along a transect through an Arctic fjord. There, a gradient in different environmental parameters was expected associated with the decreasing influence of terrestrial run-off from the mouth of the glacier towards open Arctic waters and from surface to deep waters. Additionally, surface waters were expected to experience a larger variation in the environmental parameters due to their exposure to varying meteorological conditions.

We hypothesized that surface-water bacterial communities are more variable than deep-water communities and, consequently, larger differences between the active and total bacterial community were expected in the surface as compared with the deep waters. Thus, comparison of the community structure and activity between surface (changing conditions) and deep (stable conditions) waters should provide further insight into which members of the community are potentially able to react to particular alterations in the environmental conditions.

## Results

### Composition and activity of the coastal Arctic prokaryotic community using (MICRO–)CARD–FISH

**Fig. 1:**
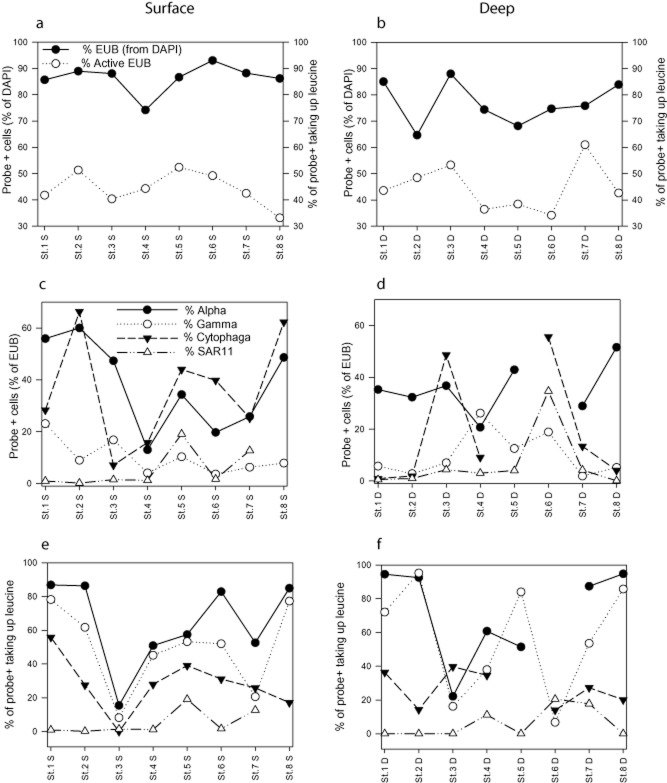
Fraction of bacteria (EUB+ cells) and proportion of bacterial cells taking up leucine detected by MICRO–CARD–FISH at the stations along the transect in Kongsfjorden as compared with DAPI-stained cells at surface (a) and deep waters (b). Percentage of specific bacterial groups (Alpha-, Gammaproteobacteria, Bacteroidetes and SAR11) detected by CARD–FISH at the stations along the transect in Kongsfjorden, Spitsbergen as percentage of the total bacterial abundance (cells hybridized with the probe mix EUB338-I to EUB338-III) (c and d), and the percentage of specific bacterial groups taking up [^3^H]-leucine at surface (e) and deep waters (f).

The prokaryotic community was dominated by bacteria (86% and 77% of DAPI-stained cells at the surface and in the deeper layer respectively; Fig. 1a and b). Euryarchaeota and Thaumarchaeota were found only in low abundance, contributing, on average, 1% and 0.6% to the DAPI-stained cells respectively (data not shown).

Alphaproteobacteria were the most abundant group accounting, on average, for 34 ± 19% at the surface and 36 ± 13% in the deeper layers to the total bacterial abundance (Fig. 1c and d). Bacteroidetes also accounted for a large fraction of the bacterial community (on average 28 ± 23% of bacterial abundance), with a higher contribution in surface than in deep waters at six out of the eight stations (Fig. 1c and d). The abundance of Gammaproteobacteria ranged between 2% and 26% of total bacterial abundance, with no clear trend in its spatial distribution along the transect (Fig. 1c and d).

Within the Alphaproteobacteria, we determined the abundance of two specific groups: SAR11 and Roseobacter. The contribution of these two groups to bacterial abundance was only minor with Roseobacter being under the detection limit at the majority of the stations (data not shown). The SAR11 clade was present at low abundance at the stations located close to the glacier and increased in its contribution to up to 36% of bacterial abundance towards the mouth of the fjord (Fig. 1c and d).

The cell-specific leucine uptake was investigated by MICRO–CARD–FISH. The fraction of bacterial cells taking up leucine (EUB probe mix-positive cells) was not significantly different between the surface and deep samples (Mann–Whitney *t*-test, *P* = 0.92; Fig. 1a and b). The members of the Alpha- and Gammaproteobacteria exhibited a high percentage of cells taking up leucine averaging 68 ± 26% and 51 ± 29% of probe+ cells respectively (Fig. 1e and f). The percentage of Alpha- and Gammaproteobacteria taking up leucine was not significantly different between surface and deep waters (Mann–Whitney *t*-test, *P* = 0.23 and *P* = 0.64 respectively) (Fig. 1e and f). Furthermore, the activity of the Alpha- and Gammaproteobacteria was significantly correlated in the surface waters (Spearman rank correlation *r*_s_ = 0.92, *P* < 0.01; Fig. 1e) but not in deep waters (Fig. 1f).

The percentage of Bacteroidetes taking up leucine was low (25 ± 13%) (Fig. 1e and f). Also, SAR11 was characterized by a low percentage of cells taking up leucine (11 ± 17%). However, the fraction of SAR11 taking up leucine increased towards the mouth of the fjord (Fig. 1e and f).

### Comparison between 16S rDNA and 16S rRNA clone libraries

**Fig. 2:**
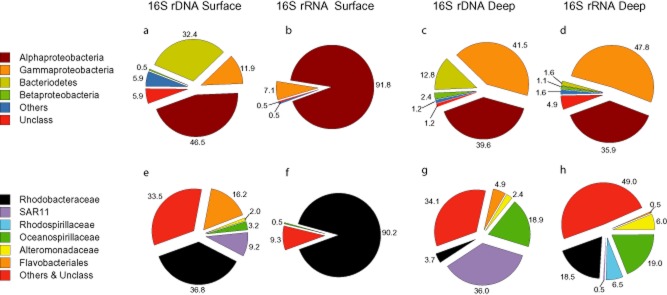
Relative contribution of (a–d) the most abundant phylogenetic classes and (e–h) families to the total number of OTUs obtained by 16S rDNA and 16S rRNA clone libraries constructed from the surface and deep waters of St. 5.

The four clone libraries (surface and deep, 16S rDNA and 16S rRNA; see supplementary material and methods) comprised in total 716 clones. The phylogenetic analysis of these clones revealed four major and four minor groups (Fig. 2). Compiling surface- and deep-water communities, significant phylogenetic differences (Unifrac significance test, *P* < 0.01) were found between the 16S rDNA and 16S rRNA clone libraries (Figs 2 and S2).

**Fig. 3:**
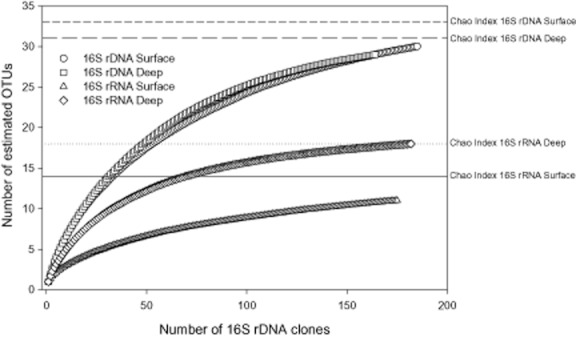
Rarefaction analysis of the clone libraries from the bacterial communities obtained at the 16S rDNA and 16S rRNA levels from surface and deep waters of St. 5. The Chao index for the OTUs sharing ≥98% identity is indicated for the respective categories by horizontal lines.

Rarefaction analyses revealed that the sequencing effort was sufficient to sample most of the members of the bacterial community given the limitations of this approach as compared with new generation sequencing (Fig. 3). The Chao richness index estimated 14 and 18 OTUs on the 16S rRNA level and 33 and 31 OTUs on the 16S rDNA level for surface and deep waters respectively (Fig. 3). Similar results were obtained with the ACE richness index (data not shown). The phylogenetic composition of the 16S rDNA and 16S rRNA clone libraries was significantly different between the two different depths as revealed by the Unifrac significance test (for both, *P* < 0.01).

The Shannon index of diversity (H′) was higher for the 16S rDNA than for the 16S rRNA in the surface waters, while in the deep waters the diversity indexes of 16S rRNA and 16S rDNA were similar (Table 1). The Pielou's evenness (J′) index showed the same trend as the Shannon index, whereas Margalef's species richness (SR) was higher for 16S rDNA than for the 16S rRNA clone libraries at both depths.

**Table 1:** Margalef's species richness (SR), Shannon index (H′) of diversity and Pielou's species evenness (J′) obtained from 16S rDNA and 16S rRNA clone libraries

Samples	SR	H′	J′
Total 16S rDNA	17.25	3.59	0.78
Total 16S rRNA	10.32	2.69	0.65
16S rDNA surface	12.07	3.34	0.80
16S rRNA surface	3.647	1.20	0.40
16S rDNA deep	11.78	3.09	0.75
16S rRNA deep	9.013	3.25	0.84

### Distribution of OTU abundance in the 16S rRNA and 16S rDNA clone libraries

The clone library of the surface-water bacterioplankton community using the 16S rRNA was dominated by one OTU that represented alone 74% of the clone library (Fig. S3); eight OTUs constituted 92% of the total bacterial 16S rRNA clone library, while singletons (i.e. OTUs occurring only once in the overall clone library) amounted to 7% of the bacterial 16S rRNA clone library. In the clone library of the surface-water bacterial community using the 16S rDNA, five OTUs represented 50% of the clone library and the singletons constituted 23% of the clone library (Fig. S3).

In the clone library of the deep-water bacterioplankton using the 16S rRNA, eight OTUs represented 50% of this clone library while the singletons accounted for 13% of the total 16S rRNA (Fig. S3). In the 16S rDNA-based clone library from deep-water bacteria, three OTUs represented ≍ 50% and the singletons 23% of the clone library (Fig. S3). Generally, the contribution of singletons was higher in the 16S rDNA than in the 16S rRNA clone libraries.

### Phylogenetic affiliation of 16S rRNA and 16S rDNA clones

The rDNA clone library from surface-water bacterioplankton was dominated by members of the Alphaproteobacteria and Bacteroidetes with 47% and 32% of the total number of clones, respectively, followed by Gammaproteobacteria (12%) and Betaproteobacteria (0.5%) (Fig. 2a). The Alphaproteobacteria class, in terms of abundance of clones, consisted mainly of the family Rhodobacteraceae (37% of the total number of clones) closely related to *Sulfitobacter*, and of SAR11 (9%) closely related to *Pelagibacter* (Fig. 2e, Table 2). The members of the Bacteroidetes class were mainly affiliated to Flavobacteriales (16% of the total number of clones) closely related to the genus *Polaribacter* (Table 2). Oceanospirillaceae was the most abundant family of the Gammaproteobacteria, amounting to 3% of the total rDNA clones (Table 2).

**Table 2:** Phylogenetic affiliation at several phylogenetic levels and the contribution of the individual families to the total number of clones obtained from 16S rDNA and 16S rRNA clone libraries in the surface and deep waters in coastal Arctic (Kongsfjorden, Ny-Ålesund, Spitsbergen)

					% of total clones	
					
	Phylum	Class	Order	Family	16S rDNA	16S rRNA
Surface	Proteobacteria	Alphaproteobacteria	Rhodobacterales	Rhodobacteraceae	36.8	90.2
	Proteobacteria	Alphaproteobacteria	Rickettsiales	SAR11	9.2	n.d.
	Proteobacteria	Gammaproteobacteria	Oceanospirillales	Oceanospirillaceae	3.2	0.5
	Bacteroidetes	Flavobacteria	Flavobacteria	Flavobacteriales	16.2	n.d.
Deep	Proteobacteria	Alphaproteobacteria	Rhodobacterales	Rhodobacteraceae	3.7	18.5
	Proteobacteria	Alphaproteobacteria	Rickettsiales	SAR11	36.0	0.5
	Proteobacteria	Alphaproteobacteria	Rhodobacterales	Rhodospirillaceae	n.d.	6.5
	Proteobacteria	Gammaproteobacteria	Oceanospirillales	Oceanospirillaceae	18.9	19.0
	Proteobacteria	Gammaproteobacteria	Alteromonadales	Alteromonadaceae	2.4	7.6
	Bacteroidetes	Flavobacteria	Flavobacteria	Flavobacteriales	4.9	0.5

n.d., not detected.

The rRNA clone library of surface-water bacteria was largely dominated by Alphaproteobacteria (92% of the total clones) followed by Gammaproteobacteria (7%) (Fig. 2b). The alphaproteobacterial family Rhodobacteraceae, closely related to the genus *Sulfitobacter*, contributed 90% to the total number of clones (Fig. 2f, Table 2).

The 16S rDNA clones from the deep-water bacterial community were dominated by Gamma- and Alphaproteobacteria with 42% and 40% of the total number of clones, respectively, followed by Bacteroidetes (13%), Betaproteobacteria (2%) and Epsilonproteobacteria (1%) (Fig. 2c). Oceanospirillaceae (19% of the total number of clones) and Alteromonadaceae (2%) were the main families of Gammaproteobacteria while the Alphaproteobacteria consisted mainly of the SAR11 clade (36% of the total number of clones) closely related to the genus *Pelagibacter*, and Rhodobacteraceae (4%) (Fig. 2g). Also the 16S rRNA clone library from the deep waters was dominated by Gammaproteobacteria (48%) and Alphaproteobacteria (36%), followed by Bacteroidetes (2%), Betaproteobacteria (1%) and Epsilonproteobacteria (1%) (Fig. 2d). The Oceanospirillaceae was again the dominant family of the Gammaproteobacteria with 19% of the total number of clones followed by Alteromonadaceae (6%), while the Alphaproteobacteria were dominated by Rhodobacteraceae accounting for 19% of the total number of clones followed by Rhodospirillaceae (7%) and SAR11 (0.5%) (Fig. 2h, Table 2).

### Contribution of different phylogenetic groups to the active and total communities

**Fig. 4:**
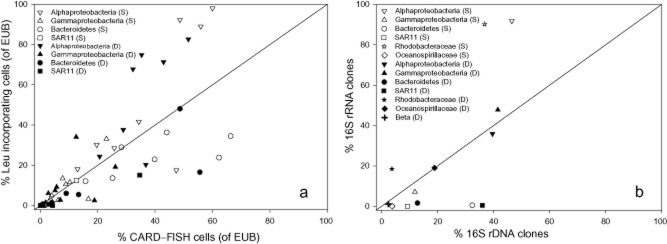
Relative contribution of specific bacterial groups to the bulk bacterial abundance determined by (a) CARD–FISH versus the percentage of cells of the respective bacterial group taking up leucine assessed by MICRO–CARD–FISH (determined in the whole transect) and by (b) 16S rDNA versus 16S rRNA clone libraries (determined at St. 5). S denotes surface samples and D deep-water samples.

The contribution of Alphaproteobacteria to the total number of bacteria taking up leucine as determined by MICRO–CARD–FISH was higher than their contribution to the total bacterial abundance (Fig. 4a). The percentage of Gammaproteobacteria taking up leucine, however, was largely proportional to their contribution to the total bacterial abundance (Fig. 4a). In contrast to Alphaproteobacteria, Bacteroidetes and SAR11 contributed disproportionally less to the bacterial community taking up leucine than to total bacterial abundance (Fig. 4a).

In the surface waters, Alphaproteobacteria and especially the members of the Rhodobacteraceae contributed relatively more to the 16 rRNA clone library than to the 16 rDNA library (Fig. 4b). The contribution of Gammaproteobacteria to the 16S rRNA library was proportional to their contribution to the 16S rDNA library in the surface waters (Fig. 4b). In the deep waters, Alpha- and Gammaproteobacteria contributed proportionally to both the 16S rRNA and 16S rDNA libraries (Fig. 4b). Bacteroidetes and SAR 11 contributed substantially less to the 16S rRNA than to the 16S rDNA clone library in both surface and deep waters (Fig. 4b). Overall, Rhodobacteraceae, closely related to *Sulfitobacter*, contributed more to the 16S rRNA clone library than other bacterial groups in both surface and deep waters (Fig. 4b).

## Discussion

During the transition period between spring and summer, the physicochemical characteristics of the water column drastically change, leading to associated alterations of biological parameters in the coastal Arctic Ocean. Two main factors drive these changes in the environmental characteristics of the water column, time and depth, both resulting mainly in temperature changes (Fig. S4) (De Corte *et al*., 2011).

### Bacterial community composition and activity assessed by MICRO–CARD–FISH

The low contribution of archaeal cells in Kongsfjorden during the spring to summer transition period (∼ 1% of the total picoplankton abundance) agrees with previous reports on the decrease of the contribution of Archaea to the prokaryotic community from the winter towards the summer season in Arctic and Antarctic waters (Church *et al*., 2003; Alonso-Saez *et al*., 2008). The active bacterial community was dominated by members of the Alphaproteobacteria, followed by Gammaproteobacteria and Bacteroidetes in agreement with earlier studies conducted in other marine environments (Morris *et al*., 2002; Cottrell and Kirchman, 2003; Longnecker *et al*., 2006; Zhang *et al*., 2006; Alonso-Saez *et al*., 2008).

The different spatial and vertical patterns displayed by the different phylogenetic groups of bacteria studied here (Fig. 1) can be explained by the different environmental conditions and their degree of variation throughout the transect. The increase of the proportion of members of Alphaproteobacteria at the offshore stations was related to the increase in abundance and activity of SAR11 towards open waters (Fig. 1) and thus supports the general notion that SAR11 is relatively more abundant under oligotrophic conditions (Morris *et al*., 2002). The components of Alphaproteobacteria responsible for their higher contribution to the bacterial community towards the inner stations could not be resolved by CARD–FISH.

Although MICRO–CARD–FISH provides information on the abundance and activity of different bacterial groups, its inherent limitations, i.e. sufficient abundance required to allow enumeration (Bouvier and del Giorgio, 2003), preclude an in-depth characterization at lower phylogenetic levels, required to more specifically link environmental conditions and prokaryotic community composition. To overcome this limitation, clone libraries were constructed.

### Bacterial community composition and activity assessed by clone libraries

Clear differences between the composition of the 16S rDNA and 16S rRNA bacterial clone libraries were found, in agreement with previous studies (Moeseneder *et al*., 2005; Gentile *et al*., 2006). These differences in the composition of the 16S rDNA and 16S rRNA clone libraries have been interpreted to result from differences between the active, detected by 16S rRNA analysis, and the total community, revealed by 16S rDNA libraries (Moeseneder *et al*., 2005). The 16S rDNA clone libraries exhibited a higher diversity than the 16S rRNA clone libraries originating from the same samples (Table 1), indicating that only a fraction of the bacterial community is active (Gentile *et al*., 2006).

The majority of the 16S rDNA and 16S rRNA clones obtained in the coastal Arctic waters were related to psychrophilic or ubiquitous phylotypes, previously found in Arctic and Antarctic marine environments (Bano and Hollibaugh, 2002; Brinkmeyer *et al*., 2003; Zaballos *et al*., 2006; Malmstrom *et al*., 2007; Pommier *et al*., 2007).

Taken together, even though the main bacterial phylogenetic groups (phylum level) are similar in surface (influenced by freshwaters originated by ice melting and terrestrial inputs, and by more variable environmental conditions) and the deep waters (characterized by more stable conditions), the community composition was significantly different in the two environments at lower phylogenetic levels, as assessed by cloning and sequencing.

### MICRO–CARD–FISH versus 16S rDNA and 16S rRNA clone libraries to assess bacterial community composition and the fraction of active bacteria

**Fig. 5:**
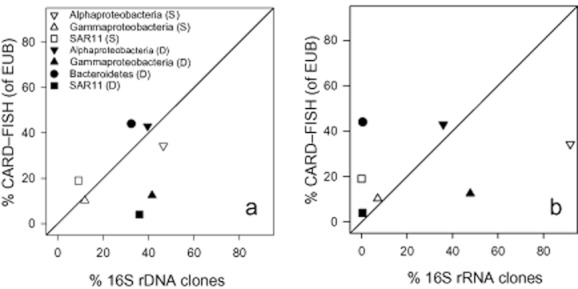
Relative contribution of specific bacterial groups to the bacterial community assessed by (a) 16S rDNA clone libraries versus CARD–FISH, and by (b) 16S rRNA clone libraries versus CARD–FISH at St. 5. S denotes surface samples and D deep-water samples.

A good agreement between the composition of total and active bacterial groups was found with both techniques (Fig. 4), indicating that 16S rRNA clone libraries are suitable to characterize the active bacterial community. However, some differences were found between the contribution of specific bacterial groups to the total community assessed by MICRO–CARD–FISH and clone libraries (Fig. 5). The low contribution of the two Alphaproteobacteria subgroups (Roseobacter and SAR11) to the bulk bacterial community determined by MICRO–CARD–FISH was not paralleled in the clone libraries where Rhodobacteraceae and SAR11 accounted for a high contribution (21% and 22% of the total DNA sequences respectively) to the total Alphaproteobacteria (Fig. 2e–h). To evaluate the discrepancy between MICRO–CARD–FISH and clone libraries, SAR11 and Roseobacter probes were matched against the 16S rDNA clone sequences. The Roseobacter probe used in this study covered 36% of the identified clones from the Rhodobacteraceae group and only 8% of the total DNA clones. Thus, more than 64% of the Roseobacter group is not covered by the Ros537 probe. Bacteria from the Roseobacter RCA clade (Selje *et al*., 2004) preferentially live in temperate and polar regions, supporting the emerging view that the global distribution of marine bacterioplankton is related to the environmental and biogeochemical properties of the water masses. Thus, Arctic assemblages are distinct from other oceanic communities and may contain autochthonous phylogenetic groups adapted to live under polar conditions (Malmstrom *et al*., 2007). This distinct phylogenetic composition of the Arctic assemblages might explain the low coverage found for the Ros537 probe against the Arctic Rhodobacteraceae clones. This low affinity results in a low number of Roseobacter cells detected by CARD–FISH in Kongsfjorden.

In contrast, the mix of SAR11 oligonucleotide probes covered > 98% of the Rickettsiales group and 21% of the total DNA clones. Thus, the discrepancy between the two methods cannot be explained by the low affinity of the oligonucleotide probe to the target group. One possible explanation is the frequently reported overestimation of the proportion of SAR11 in clone libraries (Pham *et al*., 2008). Despite this discrepancy for the SAR11 and Roseobacter subgroups, the contribution in abundance of the main bacterial groups versus their contribution to overall activity is generally comparable (Fig. 4).

Furthermore, the different proportions of active cells in specific groups determined by the two different methods might be related to the distinct metabolic targets and detection thresholds of the two methods. Some phylogenetic groups such as Alphaproteobacteria, Gammaproteobacteria and Bacteroidetes are highly diverse, and different subgroups can be specialized to utilize different substrates (Teeling *et al*. 2012) and thus might be stimulated under specific environmental conditions. The characterization of their activity by MICRO–CARD–FISH might therefore be inconclusive. In this case, cloning and sequencing, or next generation sequencing, can resolve the deeper phylogenetic affiliation of the active community, even though they are not as accurate as CARD–FISH for quantification. However, single cells can express a wide range of different metabolic and physiological states, and thus single cell activity from a specific phylogenetic group, such as SAR11, can be quantified by MICRO–CARD–FISH (Sintes and Herndl, 2006), while cloning and sequencing does not allow the discrimination of cells from the same phylogenetic group in different physiological states. Consequently, the discrepancies obtained between the two methods reflect the complexity of cellular metabolism in bacterial assemblages responding to specific environmental conditions. Our data support the notion that the physiological state of the bacterioplankton community is mainly regulated by environmental stimuli and genetic diversity, which consequently influence the cell's response (Smith and del Giorgio, 2003; del Giorgio and Gasol, 2008).

Additionally, the relative abundance obtained by CARD–FISH for the three main bacterial phylogenetic groups (Alphaproteobacteria, Gammaproteobacteria and Bacteroidetes) accounted for more than 100% of the EUB-positive cells at St. 2 and 8 in the surface layers. This unrealistically high recovery efficiency might be explained by some unspecific hybridization of the oligonucleotide probes (Bouvier and del Giorgio, 2003).

In conclusion, our results suggest that the combined use of 16S rDNA and 16S rRNA clone libraries and MICRO–CARD–FISH allows obtaining a refined view on the composition of the microbial community and its potentially active fraction, and overcomes the bias caused by either method to determine the presence and activity of specific phylogenetic groups. This approach might be used to better explain the ecological role of the low abundant groups and their function in the biogeochemical cycle. In the light of our results, the approach of cloning both the 16S rDNA and 16S rRNA and subsequent sequencing provides in-depth information on the phylogenetic affiliations of the bacterial community and its active fraction, while the MICRO–CARD–FISH allows for more accurate quantification of the members of a specific target group and their activity.

## Supplementary material

Supplementary material and methods.

**Fig. S1.** Map of the sampling sites with St. 1–8 (indicated by dots) at the Kongsfjorden, Ny-Alesund (Spitsbergen, shown in the inset).

**Fig. S2.** Phylogenetic tree based on (a) 16S rDNA and (b) 16S rRNA sequences obtained from the surface (green bars) and deep waters (blue bars). The NCBI database sequences are in black. One representative of OTUs ≥ 98% identity is shown. The bars in the two panels are scaled to the maximum number of OTUs for DNA (a, 47) and RNA (b, 136) sequences. Bootstrap values (> 80%) are indicated by the grey circles at branch point. Branch colour indicates class association; label colour indicates depth.

**Fig. S4.** Development of temperature and prokaryotic abundance (PA) and heterotrophic production PHP (in pmol leucine incorporation l^−1^ h^−1^) during the spring to summer transition (June–July 2008) in the surface and deep waters at Station St. 5. The sampling date is indicated by a frame.

**Table S1.** Physical and chemical characteristics at different stations and depths in the Kongsfjorden (Ny-Alesund, Spitsbergen, Norway).
