# Supplementary figures and images for: Comparison between MICRO–CARD–FISH and 16S rRNA gene clone libraries to assess the active versus total bacterial community in the coastal Arctic
Source: Environ Microbiol Rep. 2012 Dec 20;5(2):272–81. doi: 10.1111/1758-2229.12013 (PMC3615173; doi:10.1111/1758-2229.12013)

Figure S1

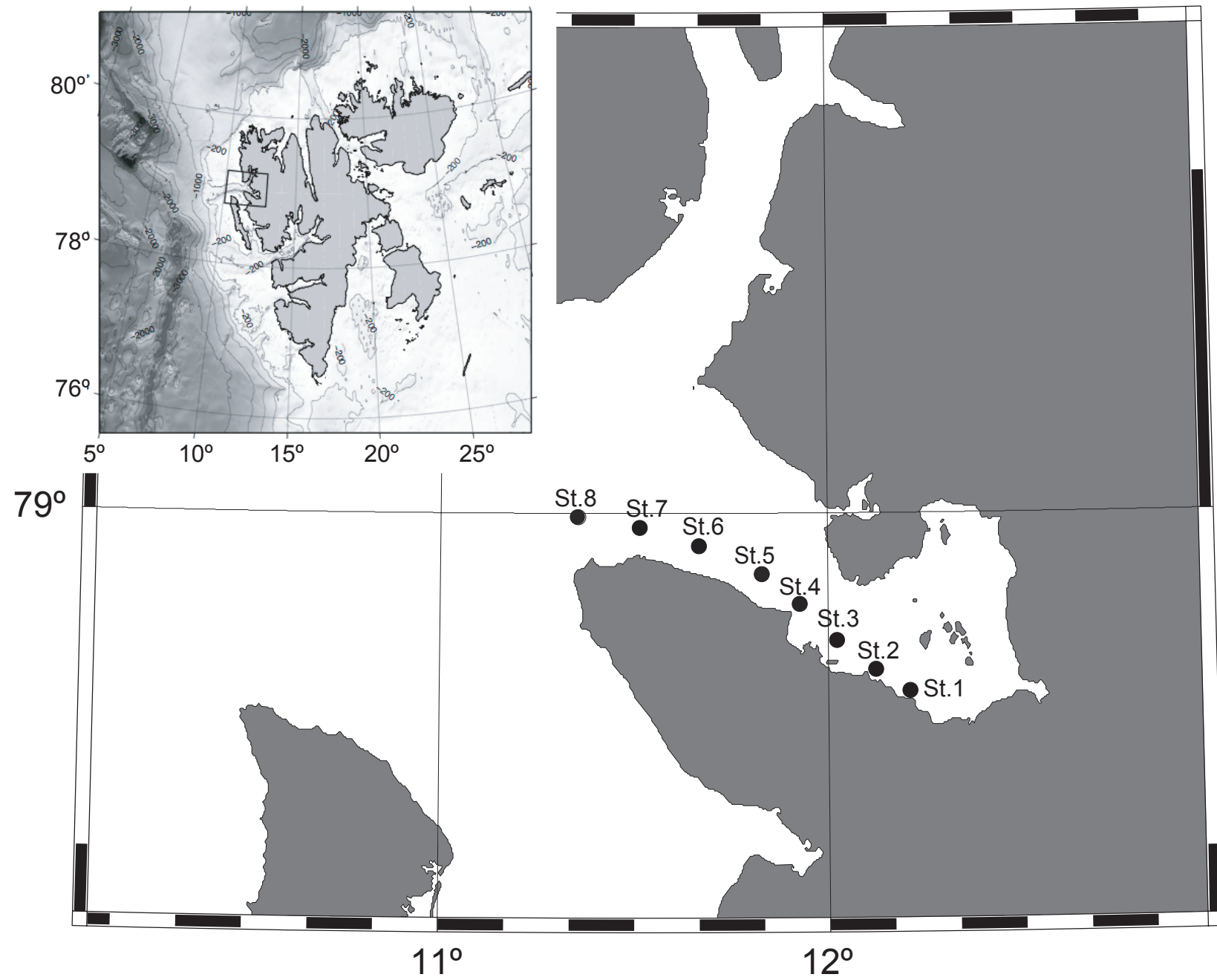

Supplement: Supplementary file 1 [file emi40005-0272-SD1.pdf]

Figure S2

a

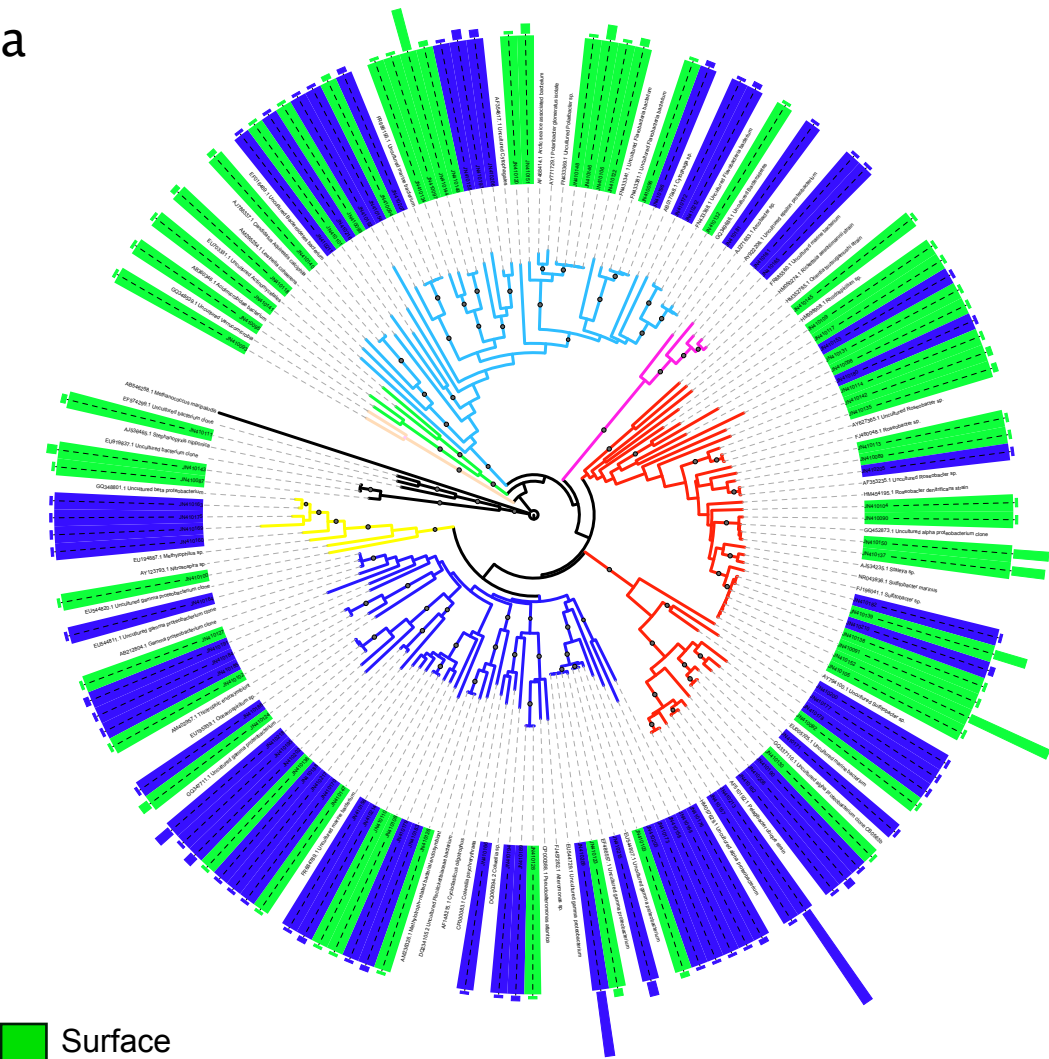

b

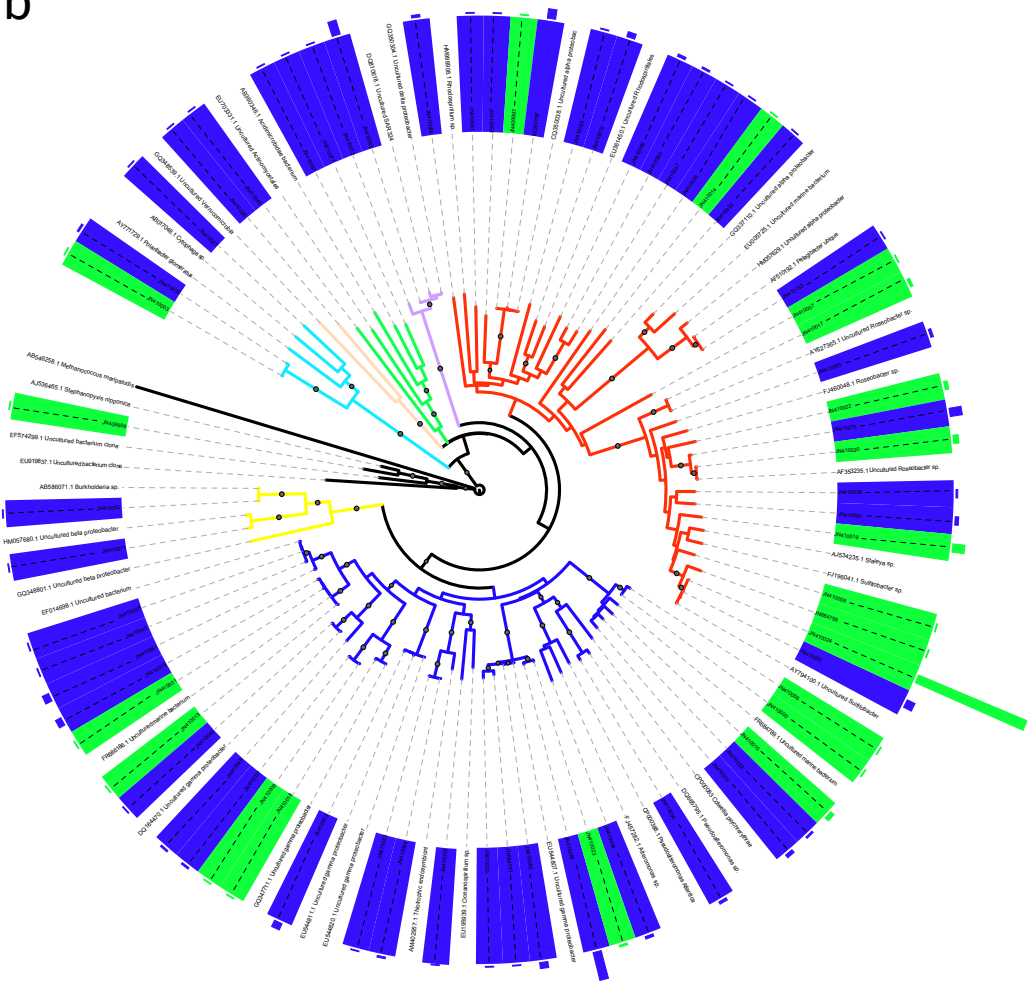

Supplement: Supplementary file 2 [file emi40005-0272-SD2.pdf]

Figure S3

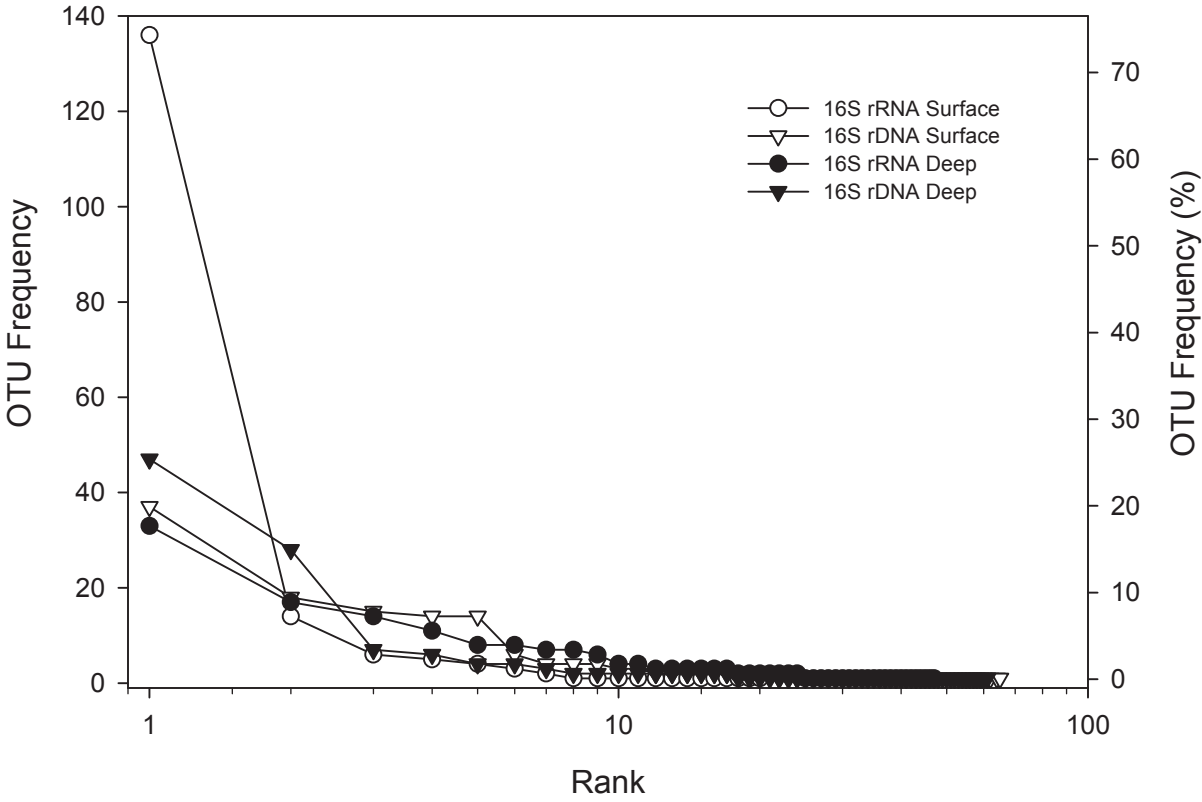

Supplement: Supplementary file 3 [file emi40005-0272-SD3.pdf]

Figure S4

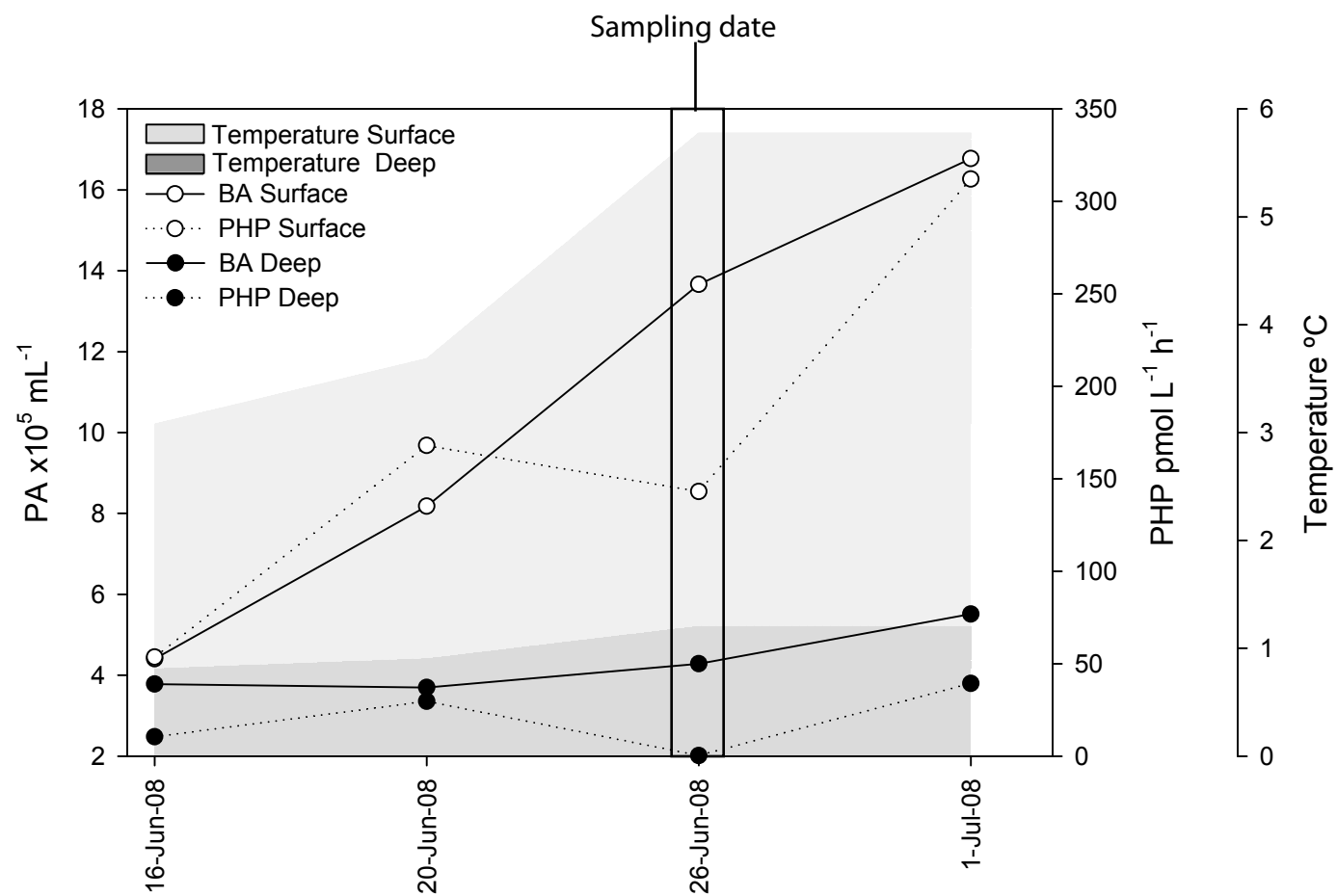

Supplement: Supplementary file 4 [file emi40005-0272-SD4.pdf]
