# Supplementary material for: Comparison between MICRO–CARD–FISH and 16S rRNA gene clone libraries to assess the active versus total bacterial community in the coastal Arctic
Source: Environ Microbiol Rep. 2012 Dec 20;5(2):272–81. doi: 10.1111/1758-2229.12013 (PMC3615173; doi:10.1111/1758-2229.12013)
Supplement: Supplementary file 5 [file emi40005-0272-SD5.doc]

Table S1. Physical and chemical characteristics at different stations and depths in the Kongsfjorden (Ny-Alesund, Spitsbergen, Norway)

|  |  |  |  |  |  |  |  |
| --- | --- | --- | --- | --- | --- | --- | --- |
| Station | Depth | Temp | Sal | NH_4_ | NO_2_ | NO_3_ | PO_4_ |
|  | (m) | (ºC) |  | µmol L^-1^ | µmol L^-1^ | µmol L^-1^ | µmol L^-1^ |
| 1 | 1.5 | 3.694 | 33.97 | 1.09 | 0.045 | 1.275 | 0.19 |
| 1 | 50 | 2.018 | 34.76 | 1.95 | 0.042 | 1.328 | 0.28 |
| 2 | 1.5 | 4.164 | 34.40 | 0.37 | 0.026 | 0.314 | 0.10 |
| 2 | 100 | 1.750 | 34.79 | 2.98 | 0.060 | 2.430 | 0.42 |
| 3 | 1.5 | 5.010 | 34.24 | 0.30 | 0.048 | 0.312 | 0.10 |
| 3 | 200 | 1.532 | 34.83 | 3.74 | 0.077 | 3.533 | 0.52 |
| 4 | 1.5 | 5.135 | 33.95 | 0.34 | 0.127 | 0.303 | 0.09 |
| 4 | 300 | 0.818 | 34.88 | 2.54 | 0.208 | 8.092 | 0.71 |
| 5 | 1.5 | 3.961 | 34.56 | 0.32 | 0.171 | 0.239 | 0.10 |
| 5 | 270 | 0.956 | 34.87 | 2.46 | 0.130 | 7.180 | 0.66 |
| 6 | 1.5 | - | - | 0.47 | 0.021 | 0.189 | 0.10 |
| 6 | 270 | - | - | 2.56 | 0.154 | 7.496 | 0.68 |
| 7 | 1.5 | 3.727 | 34.61 | 0.31 | 0.107 | 0.043 | 0.11 |
| 7 | 300 | 1.028 | 34.87 | 2.52 | 0.154 | 7.886 | 0.66 |
| 8 | 1.5 | 4.342 | 34.29 | 0.25 | 0.075 | 0.185 | 0.09 |
| 8 | 270 | 0.997 | 34.91 | 2.62 | 0.137 | 7.133 | 0.67 |

-, not measured
