# Supplementary material for: Comparison between MICRO–CARD–FISH and 16S rRNA gene clone libraries to assess the active versus total bacterial community in the coastal Arctic
Source: Environ Microbiol Rep. 2012 Dec 20;5(2):272–81. doi: 10.1111/1758-2229.12013 (PMC3615173; doi:10.1111/1758-2229.12013)
Supplement: Supplementary file 6 [file emi40005-0272-SD6.doc]

**SUPPLEMENTARY TEXT S1**

**Material and methods**

**Study area and sampling**

Water samples were taken at eight stations located in Kongsfjorden (Ny-Ålesund, Spitsbergen, Norway) following a transect from the mouth of a glacier towards open waters (Fig. S1). The samples were obtained from the surface waters (~1.5 m depth) and from a maximum depth of ~300 m during the ice melting season (June 2008). Sampling was performed from a boat with a CTD rosette sampler holding three 10-L Niskin bottles and sensors for conductivity, temperature and depth.

**Microautoradiography combined with catalyzed reporter deposition fluorescence in situ hybridization (MICRO-CARD-FISH)**

Samples for MICRO-CARD-FISH were taken along the whole transect at two depths (Table S1) between 26 and 27 June 2008. Four mL water samples were spiked with [^3^H]-leucine (specific activity: 160 µCi mmol^-1^, Amersham; final concentration 20 nM), incubated at in situ temperature (4ºC for the surface and 2ºC for the deep) for 4 h and subsequently, fixed with formaldehyde (2 % final concentration w/v) and stored at 4ºC for 24 h. The samples were subsequently filtered onto 0.2 μm white polycarbonate filters (Millipore) using a 0.45 µm cellulose nitrate supporting filter (Millipore HAWP). Processing of the filters followed established protocols (Pernthaler et al 2002a, b, Teira et al 2004). Briefly, after permeabilization with lysozyme for Bacteria and proteinase-K for Archaea (10 mg mL^-1^, 37°C for 1h), the filters were cut into sections for hybridization with the following oligonucleotide probes: a mix of Eub338-I (Amann et al 1990), Eub338-II, and Eub338-III (Daims et al 1999) to target Bacteria, Non338 (Amann et al 1995) as a control for unspecific hybridization, ALF968 (Neef 1997) for Alphaproteobacteria, GAM42a for Gammaproteobacteria (Manz et al 1992), CF319a for Bacteroidetes (Manz et al 1996), a mix of SAR11-152R, SAR11-441R, SAR11-542R and SAR11-732R targeting the SAR11 cluster (Morris et al 2002), and ROS537 (Eilers et al 2001) targeting the Roseobacter-Sulfitobacter-Silicibacter clade. A mix of Cren537 and Cren554 was used to target Thaumarchaeota (previously coined marine Crenarchaeota Group I, MCGI) while Eury806 was used to target Euryarchaeota Group II (De Corte et al 2009). The horseradish peroxidase-labeled probes were added at a final concentration of 2.5 ng μL^-1^, and hybridization was performed at 35°C for 12 to 15 h. After the washing steps, amplification was carried out by adding H_2_O_2_ and tyramide-Alexa488 and incubating the filters at 37°C for 30 min (Pernthaler et al 2002a). Slides were examined under a Zeiss Model Axioplan-2 epifluorescence microscope equipped with a 100-W Hg-lamp and appropriate filter sets for DAPI and Alexa488. Unspecific cell hybridization with the non-sense probe (Non-338) was ≤ 0.4% from total DAPI-stained cells. The contribution of Bacteria and Archaea to the microbial community was estimated as the percentage of cells hybridized by the EUB338 probe mix (Bacteria) or of the cells hybridized by the Thaumarchaeota probe mix and Eury806 probe (Archaea) as compared to the DAPI-stained cells. The relative contribution of the specific bacterial groups to Bacteria was calculated as the percentage of cells hybridized by the respective probe as compared to the EUB-probe mix positive cells.

The microautoradiography was performed following the protocol of Alonso and Pernthaler (2005). The probe-hybridized filters were glued onto glass slides and embedded in photographic emulsion (KODAK NTB-2) containing 0.1% agarose, and stored in the dark at 4°C for 48 h. The slides were subsequently developed and fixed using the specifications of the manufacturer. Thereafter, the slides were dried in a desiccator overnight and stained with a DAPI (4',6-diamidino-2-phenylindole) mixture (5.5 parts Citifluor, 1 part Vectashield, and 0.5 part phosphate-buffered saline with DAPI, 4',6-diamidino-2-phenylindole, at a final concentration of 1 μg mL^-1^), and the bacterial cells counted under a Zeiss Axioplan-2 epifluorescence microscope at 1250x magnification. The presence of silver grains around the cells was checked by switching to the transmission mode of the microscope. The proportion of cells from specific bacterial groups actively taking up leucine was determined as the percentage of probe-hybridized cells surrounded by a silver grain halo as compared to the total amount of cells hybridized. More than 200 leucine-positive cells or more than 800 DAPI-stained cells were counted per sample.

**Nucleic acids extraction and complementary DNA (cDNA) synthesis**

Fifteen L of seawater were collected at St. 5 at 1.5 m and 270 m on 26 June 2008 and filtered onto 0.2 μm polycarbonate filters (142 mm diameter, Whatman Nuclepore). Station 5, located approximately in the central part of the transect, was selected for the nucleic acid extraction as a representative station of the fjord, with surface waters influenced by the melting ice and terrestrial run-off (Table S1) and deep waters characterized by more stable conditions (De Corte et al. 2010). The changes on the environmental and biological parameters during the spring to summer transition (June-July 2008) in the surface and deep waters at Station 5 are illustrated in Fig. S2. After filtering the seawater onto the filters, 5 mL of [2-mercaptoethanol](http://www.google.it/url?sa=t&source=web&cd=1&ved=0CBgQFjAA&url=http%3A%2F%2Fen.wikipedia.org%2Fwiki%2F2-Mercaptoethanol&ei=1DFpTL2HKKeR4galgamZBA&usg=AFQjCNFUxIPEfwSQ1XLnz3lx4XfxnF2tDA) was added and the filters stored in sterile centrifuge vials at -80°C until further processing. The total nucleic acids (DNA and RNA) were extracted from the filters using the phenol extraction protocol (Weinbauer et al 2002). One fraction was kept at -80ºC for subsequent DNA analysis, while the other fraction was used to extract RNA. The RNA was isolated from the total nucleic acids by DNAase digestion (Deoxyribonuclease I, amplification grade, Invitrogen) at 37°C for 30 min. The RNA concentration was determined spectrophotometrically using a NanoDrop ND-1000 spectrophotometer (NanoDrop Technologies). The quality of the DNA and RNA extracts was checked on 1 % agarose gel after ethidium bromide staining. The efficiency of the DNA removal was tested by PCR reactions using bacterial primers. Superscript III First-Strand Synthesis supermix (Invitrogen) was used to produce cDNA from the RNA extract following the protocol of the manufacturer. The DNA, RNA and the cDNA were stored at -80ºC until further processing.

**Cloning and sequencing**

The bacterial 16S rRNA gene and 16S rRNA were amplified using the bacterial primer 27F (5’-AGA GTT TGA TCC TGG CTC AG-3’) (Lane 1991) and the universal primer 1492R (5’-GGT TAC CTT GTT ACG ACT T-3’) (Turner et al 1999). One µL of extracted nucleic acids (DNA or cDNA) was used as a template in a PCR mixture consisting of 0.3 µL of each primer, 5 µL of dNTPs, 0.3 µL of Taq polymerase Biotherm (Genecraft) and the corresponding buffer, made up to 50 µL with UV-treated ultra-pure water (Sigma). Samples were amplified by an initial denaturation step at 94ºC (for 3 min), followed by 35 cycles of denaturation at 94ºC (for 1 min), annealing at 55ºC (for 1 min), and an extension at 72ºC (for 1 min). Cycling was completed by a final extension at 72ºC for 7 min. The quality of the PCR products was checked on 1.0 % agarose gel. The PCR products were purified with QuickClean 5M PCR purification Kit (Genscript) and cloned with the TOPO-TA cloning kit (Invitrogen) according to the manufacturer’s instructions. Sequencing was performed at MACROGEN Inc. (Korea) using the 27F primer.

Sequence information obtained in this study was deposited in GenBank under the following accession numbers: JN409997-JN410218.

**Phylogenetic analysis**

The obtained sequences were compiled and aligned together with environmental bacterial sequences obtained from the NCBI database using Ribosomal Database Project (RDP, v10) online software (http://rdp.cme.msu.edu/). Chimera formation were checked using Pintail software (Ashelford et al 2005).

The phylogenetic tree was built using the Neighbour-Joining method and Jukes-Cantor distance matrix in Mega-4 software and drawn with iTOL (Interactive Tree Of Life, v2.1) (Letunic and Bork 2007). The phylogenetic affiliation of the bacterial 16S rDNA and 16S rRNA sequences was determined by Naïve Bayesian Classifier implemented in RDP (Wang et al 2007).

Rarefaction analysis and the Chao index were estimated using DOTUR (Schloss and Handelsman 2005). Several estimators of the richness and diversity of the bacterial community were determined for the individual samples, based on the clone libraries. The Chao index was used to estimate the expected species richness based on the rarefaction analysis (Chao 1984). Additionally, the Margalef’s index was used to provide the species richness normalized by the sample size (Margalef 1958). The Pielou’s evenness index was calculated to estimate the distribution of the relative contribution of OTUs to the different communities (Pielou 1975). Finally, the Shannon index was used to determine the diversity of the studied population (Chao and Shen 2003). The Margalef, Pielou and Shannon indexes were estimated using Primer-E v.6.

Operational taxonomic units (OTUs) were defined as a group of sequences differing by less than 2 % as determined by the decrease redundancy software (http://[www.expasy.org](http://www.expasy.org/)). The 98 % identity was chosen to discriminate OTUs (Stackebrandt and Ebers 2006). The phylogenetic composition of microbial communities (based on the 16S rDNA and 16S rRNA) was compared using Unifrac (Lozupone and Knight 2005).

**REFERENCES**

Alonso, C., and Pernthaler, J. (2005) Incorporation of glucose under anoxic conditions by bacterioplankton from coastal North Sea surface waters. Appl Environ Microbiol 71:1709–1716

Amann, R.I., Binder, B.J., Olson, R.J., Chisholm, S.W., Devereux, R., Stahl, D.A. (1990) Combination of 16s Ribosomal-Rna-Targeted Oligonucleotide Probes with Flow-Cytometry for Analyzing Mixed Microbial-Populations. Appl Environ Microb 56: 1919-1925

Amann, R.I., Ludwig, W., Schleifer, K.H. (1995) Phylogenetic Identification and in-Situ Detection of Individual Microbial-Cells without Cultivation. Microbiol Rev 59:143-169

Ashelford, K.E., Chuzhanova, N.A., Fry, J.C., Jones, A.J., Weightman, A.J. (2005) At least 1 in 20 16S rRNA sequence records currently held in public repositories is estimated to contain substantial anomalies. Appl Environ Microb 71:7724-7736

Chao, A. (1984) Nonparametric-estimation of the number of classes in a population. Scand J Stat 11: 265-270

Chao, A., Shen, T.J. (2003) Nonparametric estimation of Shannon's index of diversity when there are unseen species in sample. Environ Ecol Stat 10:429-443

Daims, H., Bruhl, A., Amann, R., Schleifer, K.H., Wagner, M. (1999) The domain-specific probe EUB338 is insufficient for the detection of all Bacteria: development and evaluation of a more comprehensive probe set. Syst Appl Microbiol 22:434-444

De Corte, D., Yokokawa, T., Varela, M.M., Agogue, H., Herndl, G.J. (2009) Spatial distribution of Bacteria and Archaea and amoA gene copy numbers throughout the water column of the Eastern Mediterranean Sea. ISME J 3:147-158

De Corte, D., Sintes, E., Yokokawa, T., Herndl, G.J. (2011) Changes in viral and bacterial communities during the ice-melting season in the coastal Arctic (Kongsfjorden, Ny-Ålesund). Environ Microbiol 13:1827-1841

Eilers, H., Pernthaler, J., Peplies, J., Glockner, F.O., Gerdts, G., Amann, R. (2001) Isolation of novel pelagic bacteria from the German bight and their seasonal contributions to surface picoplankton. Appl Environ Microb 67:5134-5142

Lane, D.J. (1991) 16S/23S rRNA sequencing. In: Stackebrandt E, Goodfellow M (eds). Nucleic acid techniques in bacterial systematics. Wiley and Sons Ltd.: Chichester, United Kingdom

Letunic, I., Bork, P. (2007) Interactive Tree Of Life (iTOL): an online tool for phylogenetic tree display and annotation. Bioinformatics 23:127-128

Lozupone, C., Knight, R. (2005) UniFrac: a new phylogenetic method for comparing microbial communities. Appl Environ Microb 71: 8228-8235

Margalef, D.R. (1958) Information theory in ecology. General Systems Bulletin 3:36-71

Morris, R.M., Rappe, M.S., Connon, S.A., Vergin, K.L., Siebold, W.A., Carlson, C.A., Giovannoni, S.J. (2002) SAR11 clade dominates ocean surface bacterioplankton communities. Nature 420:806-810

Neef, A. (1997) Anwendung der in situ-Einzelzell- Identifizierung von Bakterien zur Populationsanlayse in komplexen mikrobiellen biozönosen. PhD Thesis. Technische Universität Munchen. Münich, Germany

Pernthaler, A., Pernthaler, J., Amann, R. (2002a) Fluorescence in situ hybridization and catalyzed reporter deposition for the identification of marine bacteria. Appl Environ Microb 68:3094-3101

Pernthaler, A., Preston, C.M., Pernthaler, J., DeLong, E.F., Amann, R. (2002b) Comparison of fluorescently labeled oligonucleotide and polynucleotide probes for the detection of pelagic marine bacteria and archaea. Appl Environ Microb 68: 661-667

Pielou, E.C. (1975) Ecological Diversity. John Wiley and Sons: New York

Schloss, P.D., Handelsman, J. (2005) Introducing DOTUR, a computer program for defining operational taxonomic units and estimating species richness. Appl Environ Microb 71:1501-1506

Stackebrandt, E., Ebers, J. (2006) Taxonomic parameters revisited: tarnished gold standards. Microbiol Today 33:152-155

Teira, E., Reinthaler, T., Pernthaler, A., Pernthaler, J., Herndl, G.J. (2004) Combining catalyzed reporter deposition-fluorescence in situ hybridization and microautoradiography to detect substrate utilization by bacteria and Archaea in the deep ocean. Appl Environ Microb 70:4411-4414

Turner, S., Pryer, K.M., Miao, V.P., Palmer, J.D. (1999) Investigating deep phylogenetic relationships among cyanobacteria and plastids by small subunit rRNA sequence analysis. J Eukaryot Microbiol 46:327-338

Wang, Q., Garrity, G.M., Tiedje, J.M., Cole, J.R. (2007) Naive Bayesian classifier for rapid assignment of rRNA sequences into the new bacterial taxonomy. Appl Environ Microb 73:5261-5267

Weinbauer, M.G., Fritz, I., Wenderoth, D.F., Hofle, M.G. (2002) Simultaneous extraction from bacterioplankton of total RNA and DNA suitable for quantitative structure and function analyses. Appl Environ Microb 68:1082-1087
